# Multi-nanolayer drug delivery using radiofrequency plasma technology

**DOI:** 10.1186/s12885-020-06989-w

**Published:** 2020-06-17

**Authors:** Iman Al Dybiat, Alibi Baitukha, Cynthia Pimpie, Rachid Kaci, Marc Pocard, Farzaneh Arefi Khonsari, Massoud Mirshahi

**Affiliations:** 1 CAP-Paris Tech, INSERM U1275, Department of Oncologic & Digestive Surgery, Université de Paris, Lariboisière Hospital, 2 rue Ambroise Paré, 75010 Paris, France; 2 Laboratoire Interfaces et Systèmes Electrochimiques, Sorbonne Universités, University Paris 06, CNRS, 4 place Jussieu, 75005 Paris, France; 3 Central Department of Anatomy and Pathological Cytology, Hospital Lariboisière, 75010 Paris, France

**Keywords:** Cancer, Drug delivery, Film implantation, Multi-nanolayer technology, Radio frequency plasma

## Abstract

**Background:**

It may be impossible to perform cancer surgery with free margins in the presence of an unresectable structure. Local drug treatment after surgery has been proposed to increase the rate of tumor control.

**Methods:**

Multi-nanolayers (10-330 nm) were generated by a low-pressure (375mTorr) inductively coupled plasma (13.56 MHz) reactor for anticancer drug delivery by the deposition of polycaprolactone-polyethylene glycol multistack barrier on the collagen membrane (100 μm thickness). Carboplatin (300 μg/cm^2^) was used for the in vitro and in vivo investigations. Energy-dispersive X-ray spectroscopy (15 keV), scanning electron microscopy and inductively coupled plasma mass spectrometry were used to detect the presence of carboplatin in the nanolayer, the tumor sample and the culture medium. Preclinical studies were performed on ovarian (OVCAR-3NIH) and colon (CT26) cancer cell lines as *xenografts (45 days)* and *allografts* (23 days) in Swiss-nude (*n* = 6) and immunocompetent BALB/cByJ mice (*n* = 24), respectively.

**Results:**

The loading of carboplatin or other drugs between the nanofilm on the collagen membrane did not modify the mesh complex architecture or the drug properties. Drugs were detectable on the membrane for more than 2 weeks in the in vitro analysis and more than 10 days in the in vivo analysis. Cytotoxic mesh decreased cell adherence (down 5.42-fold) and induced cancer cell destruction (up to 7.87-fold). Implantation of the mesh on the mouse tumor nodule modified the cell architecture and decreased the tumor size (50.26%) compared to the control by inducing cell apoptosis.

**Conclusion:**

Plasma technology allows a mesh to be built with multi-nanolayer anticancer drug delivery on collagen membranes.

## Background

Nanotechnology is one of the fastest growing scientific fields today. Several applications, such as the detection of pathogens, drugs and gene delivery for cancer therapy have been documented recently [1–5]. Because of their microsize, nanomaterials have become indispensable in many applications for human needs [6]. Chemotherapy and radiotherapy, due to their secondary side effects, continue to be limited in cancer treatment and are not the optimal solutions to face the problem of cancer. However, such therapy is the only possibility in cases with a specific tumor location. Other treatments, such as regional hyperthermia, have been proposed. Regional hyperthermia associated with chemotherapy improves local progression-free survival but not overall survival [7, 8]. Targeted therapy is a newer type of treatment by which drugs or antitumor substances are used to exert direct effects on cancer cells with little or no damage to neighboring normal cells. For these objectives, different techniques and variable preparation protocols have been formulated in wet and dry conditions [9–15], Compared with wet processes, the use of a biodegradable polymer substrate as a drug carrier seems to be a promising method for delivering anticancer drugs, especially in postoperative local chemotherapy. Plasma polymerization is a dry and single-step method that is solvent free [16]. Plasma (co-)polymerization of different organic monomers for surface modification with a variety of substrates has been used for tunable biomolecule-surface interactions and controlled drug delivery applications [17, 18]. However, the inconvenience of this method has been reported by free-radical-induced grafting [19]. In a previous work from our laboratory, we reported the development of a drug delivery system based on different layers of plasma copolymerized poly-ε-caprolactone-polyethylene glycol (PCL-PEG) copolymers [20–23].

Here, we report the development of a multi-nanolayer for drug delivery in an animal model for specific clinical and anatomical situations.

## Methods

### Nanofilm preparation

The low-pressure inductively coupled plasma (ICP) reactor is schematically illustrated in Fig. S1A. Diethylene glycol dimethyl ether (diglyme, 134.17 g/mol, C_6_H_14_O_3_, Sigma - France) and ε-caprolactone were used as the precursor materials and delivered into the reactor through the bubbling in of argon gas. The operating pressure was set to 375 mTorr and was controlled by adjusting the gate valve of the turbo-molecular pump. A 13.56 MHz Radio frequency RF power supply was programmed to deliver varying energy input into the reactor, which resulted in the deposition of a controlled sequence of layers with varying densities and thicknesses. This allowed us to automate the multistep deposition procedure to deposit composite films with different deposition parameters and to fabricate films with alternating or gradually changing densities, chemical compositions and mechanical properties. Two different monomers were used to form co-polymers, ε-caprolactone for PCL and diethylene glycol dimethyl ether (dyglime) for PEG components Fig. S1B. Carboplatin, oxaliplatin, ferrite beats and M2YN were used as the components to be encapsulated between the plasma polymerized barrier layers. The density of the loaded drug was 300 μg/cm^2^ for carboplatin and oxaliplatin, 30 μg/cm^2^ for ferrite beats and 30 μg/cm^2^ for M2YN.

### Study of the fabricated films

The films produced were incubated for 10 min in 2.5% glutaraldehyde, washed four times with distilled water and then dehydrated in increasing concentrations of ethanol. The samples were dried and then sputter-coated with gold (Cressington 108 auto/SE) or carbon (Cressington 208-carbon). The films were observed by scanning electronic microscopy (SEM) with a Zeiss Ultra 55 FEG scanning electronic microscope from 1 to 15 kV.

### Carboplatin measurement

The collagen membrane (Biom’UP COVA+) is a CE marked membrane, composed of porcine collagen that forms a barrier to provide the guided healing of organs and tissues along distinct anatomical planes [24, 25]. Collagen membrane with only a barrier coating layer was considered the control in this study. The films loaded with carboplatin were incubated in the culture medium for certain periods of time (4 h, 24 h, 72 h or 192 h) at 37 °C, 5% CO_2_ and > 80% humidity. The films and medium were collected after the experiment and prepared for measuring the carboplatin that was released into the medium and any carboplatin that was still deposited on the film. For this measurement, we checked the films and culture medium via energy-dispersive X-ray spectroscopy (Zeiss) and ICP-MS (inductively coupled plasma mass spectrometry, Elan DRCe, Perkin Elmer®), respectively.

### Cancer cell lines

The ovarian cancer cell line OVCAR-3 and the colon cancer cell line CT-26 were obtained from the American Type Culture Collection (ATCC, Manassas, VA). OVCAR-3 and CT26 cells were maintained in RPMI and DMEM medium (Gibco, Saint Aubin, France), respectively. The cellular environment was maintained at 50 mL/L CO_2_, 80% humidity and 37 °C.

### Animals

A total of 6 nude mice and 24 female BALB/c mice (4 weeks old) were purchased from Charles River Laboratories (Arbresle, France), and their body weights ranged from 20 to 25 g. All animals were maintained at the animal center for 2 weeks of adaptive feeding prior to the start of the experiment. The animals were randomly divided into two groups. The mice were caged in groups of three for the nude mice and groups of five for the BALB/c in an air-filtered laminar flow cabinet and fed with irradiated food and autoclaved reverse-osmosis treated water. All procedures were performed under sterile conditions in a laminar flow hood. Five animals from each group were used. The experimental protocol was approved by the Ethics Review Committee for Animal Experimentation of UPMC, France (*APAFiS* Number 3790). All experimental protocols were performed in accordance with the European Convention for the protection of vertebrate animals used for experimental and other scientific purposes (Council of Europe, 1986, ETS No. 123).

### Tumor production

Ovarian cancer OVCAR-3 cells and colon cancer CT26 cells (5 × 10^4^/well) were seeded on 8 type 75 flasks and incubated with the RPMI and DMEM cell culture medium, respectively, at 37 °C, 5% CO_2_ and < 80% humidity. After 72 h, the cells were collected following enzymatic treatment with 1% trypsin (Sigma, France). Two milliliters of 1% trypsin was added to each flask after washing the flask with Phosphate buffered *saline* (PBS) to avoid any interference of the medium with the enzymatic activity. The flasks were incubated with the enzyme for 2 min at 37 °C (incubator); then, 5 ml of RPMI or DMEM were added to the OVCAR-3 or CT26 flasks, respectively. The collected medium was centrifuged for 5 min at 2000 rpm. The supernatant was removed thereafter, and the cells were counted via C-ship (Dutscher, France). Then, 100 μl (10^5^ CT26 murine cells) was injected into the inguinal lymph node of each mouse after anesthetizing the mice with 2% isoflurane in oxygen with mechanical ventilation for 15 min. The position of the inguinal lymph node is described in the supplementary data Fig. S2. For the nude mice whose immune system was inhibited, 100 μl (10^6^ OVCAR-3 human cells) was injected subcutaneously. Mice were monitored for 2 to 3 weeks to check the development of the tumor nodules. Once the nodule grew to approximately 0.4 cm, implantation of the films was performed either with a control film where no drug was loaded or with carboplatin deposited on the film.

### Film implantation

In a previous work, we found the optimized amount of drug on the substrate surface of 1 cm^2^ [22]. The amount of 30 μl of Pt = 300 μg/cm^2^ film was used in this study [20]. After developing a tumor, the animals were anesthetized with 2% isoflurane. Each animal was operated on after skin disinfection with Betadine (Vetoquinol SA, France). The skin was opened approximately 2 cm where the tumor developed. A collagen implant (1 × 0.5 cm) was deposited on the tumor, and the skin was closed via stitching (Peters Surgical, France), and some drops of Betadine were applied again on the operated zone.

### Sample collection

After 10 days of implantation and to collect samples, the mice were sacrificed by cervical dislocation after being anesthetized with general gas anesthesia 4% isoflurane (Baxter- Guyancourt- France). The tumor size was measured to evaluate the efficacy of the treatment. The tumors and films were recovered after marking the contact zone of the tumor with the film. Each tumor was divided into three parts. One was for the histological analysis, where pieces were fixed with 4% PFA (Sigma, France) and kept at 4 °C until the analysis. One part was kept at − 80 °C in OCT (CellPath, UK) for the immunofluorescence study, and the third part was kept at − 80 °C to detect the presence of carboplatin in the tumor via Energy-dispersive X-ray spectroscopy EDX. Moreover, the implanted films were fixed with 4% paraformaldehyde and kept at 4 °C for scanning electron microscopy (SEM).

### Statistical study

1. Tumors from both groups (treated and control) were measured on the day of implantation, T1, and on the day of sample collection, T2. The standard error and the average absolute deviation were calculated, and the values were compared to study the increasing size of both the control and treated groups.
2. Five zones were randomly selected after Immuno-histochemical coloration IHC and SEM for the cell adhesion measurement via counting cells in both the treated and control zones. A nonparametric *(Mann*–*Whitney)* test was performed with GraphPad Software. *P*-values under 0.05 were considered significant.

### In vivo cell viability

Films were collected from both the control and treated groups. Using the scanning electron microscopy images, the cells attached to both films were counted, and an average of three readings were calculated and compared in both groups. A *Mann*–*Whitney* test was applied with GraphPad Software. Values under 0.05 were considered significant.

### Cell death analysis

To document the release of carboplatin from the film and its effects on the contact zone with the tumor cells, pieces were cut per cryostat (7 μm) at − 20 °C (Leica X). The slides were fixed with 4% PFA for 5 min and washed with 100 μl of PBS. Fluorescein-dUTP (Sigma, France) was added to each slide for 30 min, and then a drop of 4′,6-diamidino-2-phenylindole DAPI (Vector, Burlingame) was added for the mounting. The slides were checked by an EVOS® FL auto imaging system (Life Technologies™, Waltham, USA), where green spots on the slide indicated DNA fragments in necrotic cells.

### Histochemical analysis

The fixed samples were embedded in paraffin, and slides were produced (4 μm) and colored by hematoxylin-eosin-saffron according to classical methods in the anatomopathological laboratory (Lariboisiere Hospital). In parallel, the slides from the OVCAR-3 tumors were stained by antibodies coupled with peroxidase using a Benchmark Ultra apparatus (Roche, Ventana, Tucson, Arizona, USA) according to the manufacturer’s instructions. The antibodies used for the immunohistochemistry were as follows: E-cadherin, cytokeratin, Ki67 and beta-catenin (Glostrup, Denmark, and Carpinteria-California, USA). The slides were studied and pictures were taken by Leitz (Diaplan) microscopy with a Nikon Coolpix 995 apparatus (Japan). The necrotic zones were measured by ImageJ software.

## Results

### Multi-nanolayers can be deposed over the biodegradable collagen membrane

**Fig. 1:**
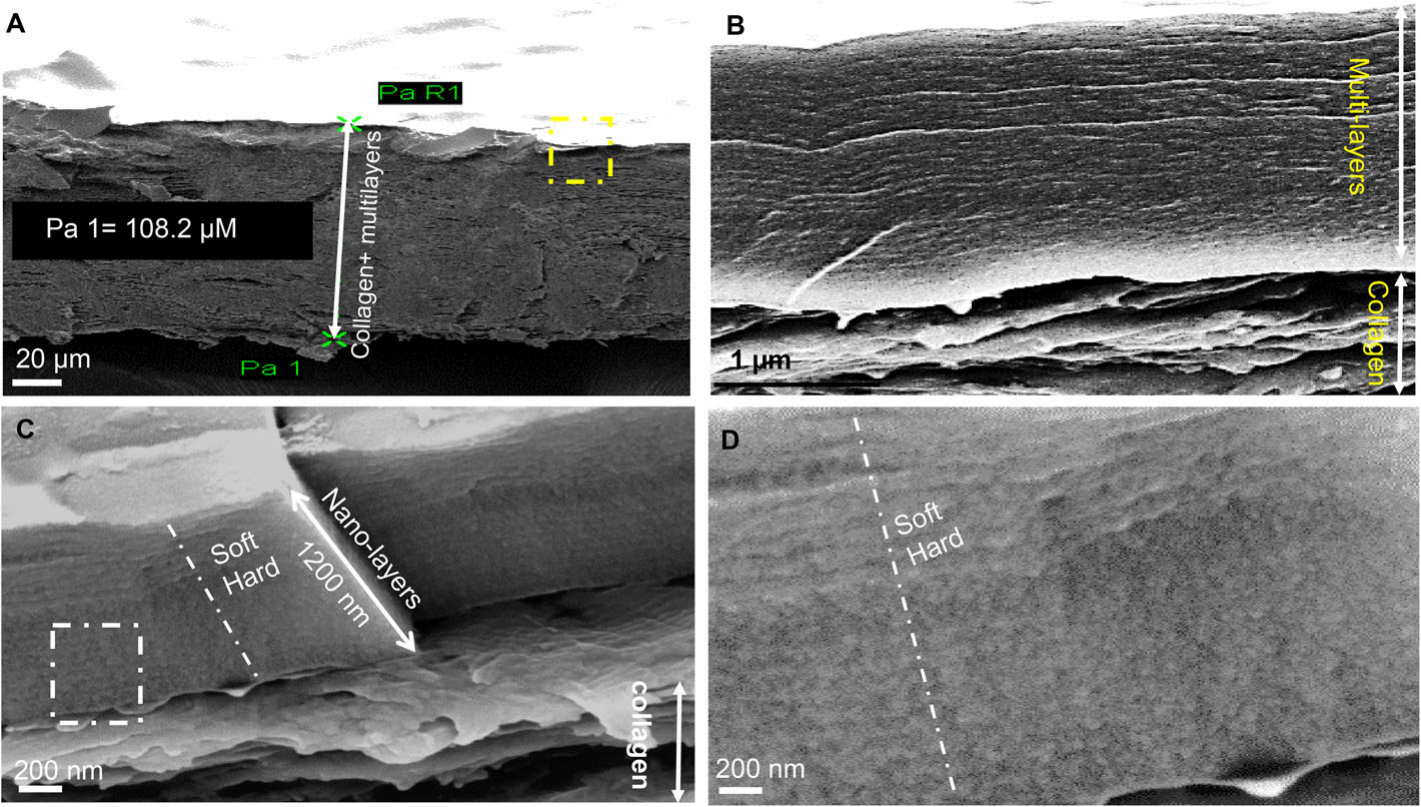
Nanolayers deposited on the collagen membrane. **a**. Generated multi nanolayers over collagen membrane 108.2 μm. **b**. Multi nanolayers with higher magnification. **c**. Alternating ‘soft’ and ‘hard’ films forming a composite barrier layer. **d**. with higher magnification

As presented in Fig. 1a and b and using the ICP reactor, we produced nanolayers deposited over the collagen patch with variable thicknesses between 10 and 1000 nm. Applying different duty cycles of the pulses during the deposition process allowed to produce copolymers with different densities. In continuous mode (100% duty cycle and an effective power of 25 W), we achieved a high level of fragmentation of the introduced precursor and deposited highly diamond-like ‘hard’ films. Additionally, pulsing at 10% of the duty cycle and an effective power of 2.5 W generated an energy-deficient deposition process resulting in the formation of a soft film Fig. 1c and d. By alternating ‘soft’ and ‘hard’ layers, we were able to deposit elastic composite films with overall good mechanical and barrier properties that helps to reduce the formation of microcracks on the film surface and a sudden release of the drug. In the photo presented, 20 soft layers and 20 hard layers were designed; each ‘hard-soft’ sequence measured 50 nm with 15 nm for each soft layer and 35 nm for each hard layer. These composite multilayer barrier films were encapsulated by the barrier film on the surface with antifouling properties, and another barrier layer was deposited directly on the collagen membrane. The latter is performed to prevent the infiltration of the drugs through the porous collagen membrane.

### Loading of carboplatin onto the nanofilm

**Fig. 2:**
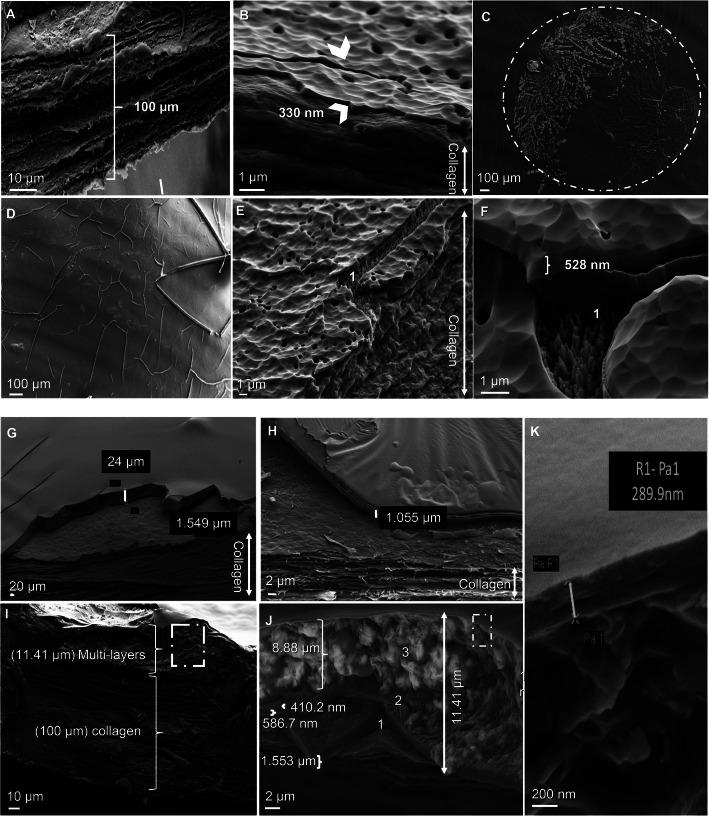
Producing biodegradable films with nanolayers. **a**. Scanning electron microscopy view, collagen layer of 100 μm. **b**. Collagen and mono/multi-nanolayers (white arrows indicate to the nanofilm deposited over the collagen membrane. **c**. Drug is loaded and encapsulated within the nanolayer (white pointed circle). **d**. Cracks over the film. **e**. Carboplatin detection throughout the film where the drug is indicated by a white number 1. **f**. Drug presence in crystal form as indicated by number 1. **g**. Oxaliplatin. **h**. M2YN. **i**. Sandwich film with many nanolayers. **j**. Layer presentation as the following: 1) Carboplatin 2) Iron beads and 3) Oxaliplatin. **k**. Nanocoating film deposition (last layer) of 290 nm. This sandwich measures 11.41 μm and the collagen measures approximately 100 μm

**Fig. 3:**
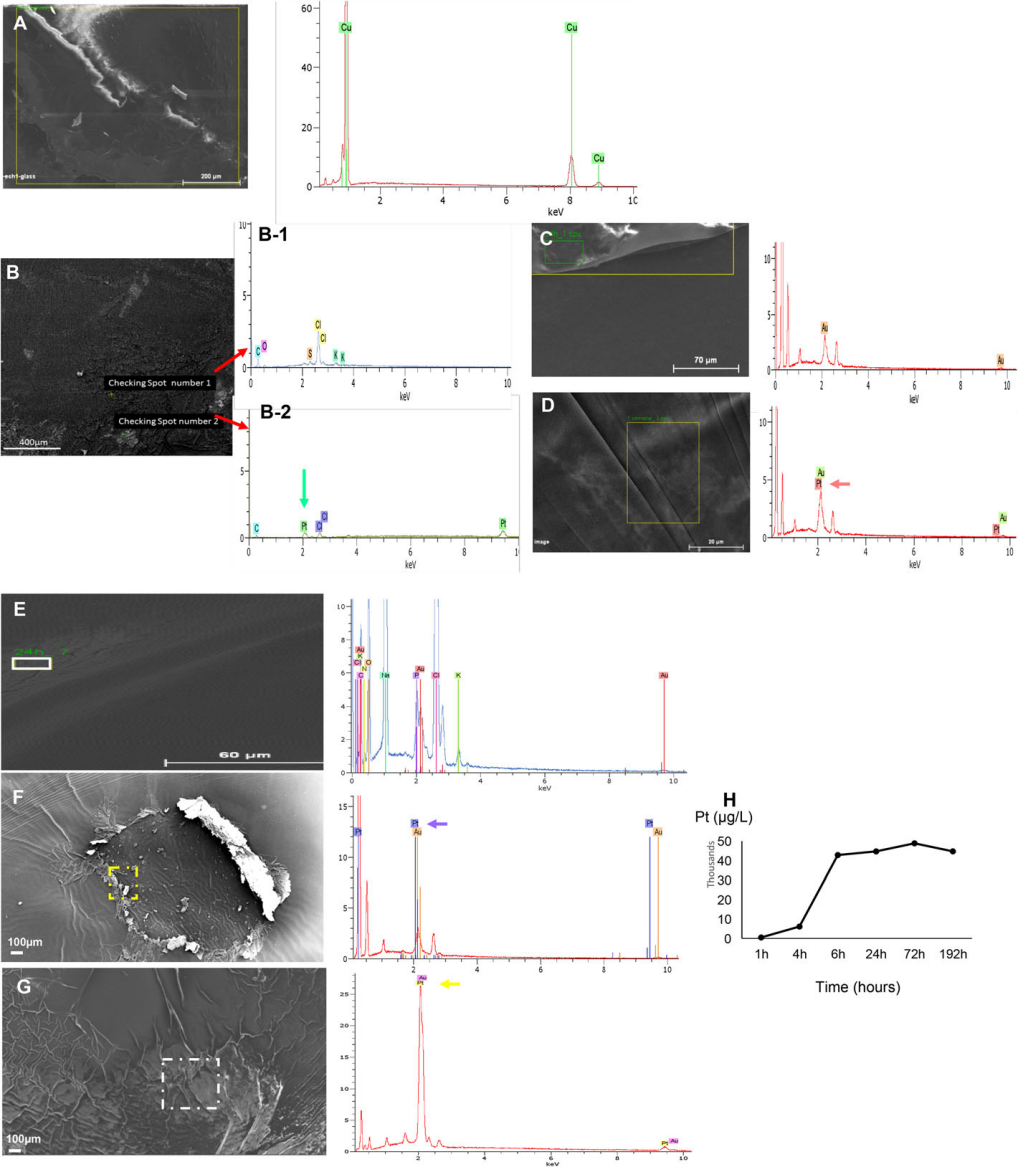
Confirmation of the presence of carboplatin loaded on the film after preparation. **a**. Control of the detector via the calibration of copper (Cu) as a reference shown in a green peak. **b**. Film with loaded drug in which two points were checked. Spot B-1 represents the control where no carboplatin was found, but the other elements detected came from the culture medium when the film was incubated: C for carbon, O for oxygen, S for sulfur, Cl for chloride and K for potassium. Spot B-2 represents carboplatin (Pt green peak). The films were covered with a carbon coat before scanning. **c**, **d**. Another experiment that performed with a gold (Au) coating layer. We were able to recognize the platinum (Pt) shown in pink peak beside the gold (Au) peak. **e**. Control film where no drug was loaded onto the film and no detected carboplatin Pt peak. K in the graph is for potassium, N is for nitrogen, C is for carbon, Cl is for chloride, O is for oxygen, Na is for sodium, P is for phosphor. F. Film loaded with drug in the presence of carboplatin after 24 h of incubation and **g**. after 8 days of incubation. The presence of carboplatin Pt is shown as Pt peak in the corresponding graphs. Y-axis (cps/eV) is counts per second per eV. X-axis (KeV) is kilo electron volts. **h**. Kinetics of the released carboplatin in the culture medium till 192 h of incubation. Yellow and white zones in **a**, **c**, **d**, **e**, **f** and **g** represent the checked areas with EDX spectroscopy

After producing the different nanolayers (330 nm) deposited on the collagen (100 μm) Fig. 2a and b, we aimed to load an anticancer drug, carboplatin (Pt), within these multilayers (30 μl of Pt = 300 μg/cm^2^ film). A view of the membrane with the encapsulated drug is presented in Fig. 2c. When this membrane cracked after exposure to liquid nitrogen, a membrane fracture was observed Fig. 2d, and the presence of the dehydrated carboplatin drug between the two layers is shown in Fig. 2e. Details of this observation are presented in Fig. 2f, where the drug was detected in its crystallized form. In addition to carboplatin, we tried to apply other materials, such as oxaliplatin (Fig. 2g) and plant polyphenol extract Fig. 3h using the same method of deposition and encapsulation within two nanolayers. A sandwich of three layers was tested, and three substrates were loaded Fig. 2i. Fig. 2j shows this in greater detail from below: 1 indicates where the carboplatin drug was deposited, 2 in the middle indicates where antibody-iron beads were deposited to facilitate a distinction between the layers, and 3 indicates the drug oxaliplatin. The last deposition was followed by a nanocoating film deposition of 290 nm, as shown in Fig. 2k. All results indicate that the ICP reactor generated a biocompatible membrane with three or more layers of drug with a thickness of 0.1 to 0.15 mm over the collagen membrane.

### In vitro study of the drugs loaded in multi-nanolayers

Our next objective was to study the physical features of the fabricated films.

#### Detection of carboplatin in the films produced

Other techniques were used to confirm the presence of deposited carboplatin onto the film and its encapsulation inside. Energy-dispersive X-ray spectroscopy (EDX) was applied to the produced films. The first step presented in Fig. 3a, was the calibration/control of the machine where copper (Cu) is usually used; this step comes before determining the presence of carboplatin or any other mineral. The film was covered by either a carbon (C) or gold (Au) layer before checking the sample to avoid an extra charge on the image. Fig. 3b shows the two checked spots of the film (B-1, B-2). In B-1, no Pt was detected, but a Pt peak was detected in B-2. The film was covered with carbon before the EDX check, which is why a carbon peak was present in both spots; the other minerals observed, such as chlorine (Cl), sulfur (S), oxygen (O) and potassium (K), belonged to the culture medium, as the films were incubated before. In Fig. 3c, the control film (without the deposited drug) was coated by gold (Au) and was presented with one peak of gold (Au) and no platinum (Pt) detected. Fig. 3d shows two peaks, platinum Pt and gold Au; the latter was deposited over the film before the EDX check.

#### Measurement of the amount of carboplatin released from the incubated film into the culture medium

After depositing carboplatin onto the film and confirming its presence in the encapsulated form within the two nanolayers, we aimed to study the kinetics of carboplatin release when incubated in the culture medium at 37 °C, similar to the in vivo conditions in the mouse model. The incubation of the films was performed for the detected points at 4 h, 24 h, 48 h, 72 h and 192 h. Each film was analyzed simultaneously by EDX to detect the presence of carboplatin in the films and by ICP-MS to detect Pt in the mediums. The results in Fig. 3e and f, g present the control and released carboplatin, respectively, from the first few hours after incubation. Drug release continued until the 7th day of incubation. Fig. 3f presents the released Pt after 24 h, and Fig. 3g shows the amount released after 8 days. The histogram in Fig. 3h shows the kinetics in the culture medium after 8 days (*P < 0.0022*).

### In vivo study of deposited carboplatin films

#### Tumor production and implantation of the drug film into mice

**Fig. 4:**
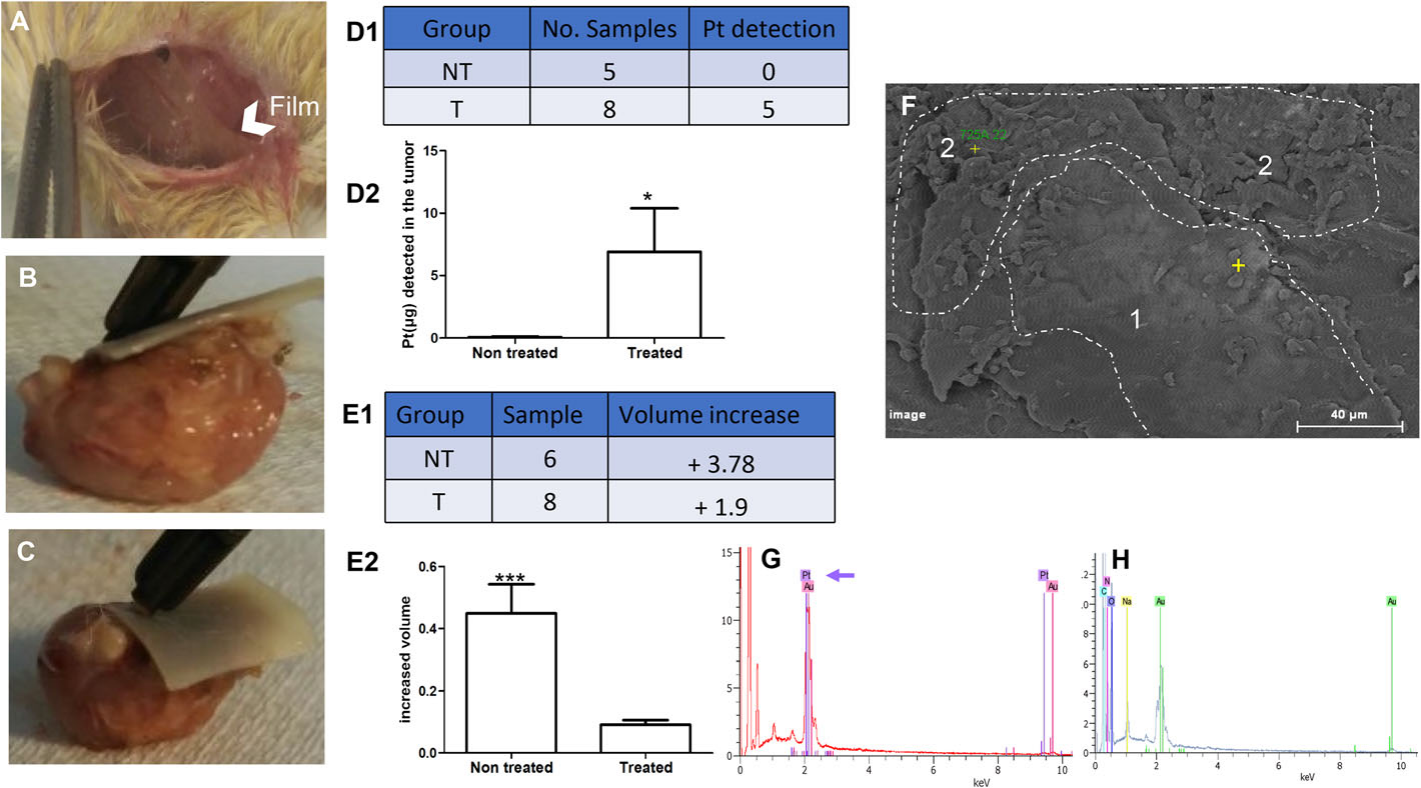
In vivo: films are applicable to the BALB/c mouse animal model. **a**. Implanted film in the BALB/c mouse model where the tumor was produced in the inguinal lymph node (white arrow indicates the film position). **b**. Sampling of the control film after implantation. **c**. Sampling of the treated film after implantation. **d**. 1- Pt detection via ICP-MS in nontreated (NT) and treated (T) mice. 2- Histogram of the NT and T groups, *P <* 0.0286. **e**. 1- Rate of decreasing volume in the treated group, T, compared with the control, NT. 2- Histogram of the NT and T groups, *P <* 0.0047. **f**. Film surface from the contact zone with the nodule was checked; SEM showed a difference in the adherence density in the presence (zone 1) or absence (zone 2) of platinum. **g**. Zone 1 checked for Pt presence (purple peak). **h**. Zone 2, where no Pt was detected. Yellow plus signs indicate to the checked area with EDX. Au in the graphs is for gold, N is for nitrogen, C is for carbon, O is for oxygen, Na is for sodium. Y-axis (cps/eV) is counts per second per eV. X-axis (KeV) is kilo electron volts

Approximately 10^5^ cells of the colon cancer cell line CT26 were injected into the inguinal lymph node of BALB/c mice (*n* = 24). The mice were monitored for 2 to 3 weeks. Once the tumor grew to a size of 0.3 to 0.5 cm, a control or drug-loaded film was implanted into each mouse and deposited over the tumor Fig. 4a. Ten days after implantation, the films and the tumors were removed together for study Fig. 4b, tumor with control film, and C, tumor with drug-loaded film). A small piece (approximately 20 mg) of the contact zone between the film and the tumor was taken from five controls and eight treated samples and checked for carboplatin detection via inductively coupled plasma mass spectrometry (ICP-MS). Carboplatin was present in five of the eight tumors of the treated mice (*P <* 0.028), whereas no carboplatin was detected in any of the five nontreated mice Fig. 4d1-2. The statistical study of the tumor volume between the day of implantation and the day of sampling showed that the control group had a 3.78-fold increase in volume from the day of implantation, while the treated group had a 1.9-fold increase in volume. Thus, the tumors without treatment had two times more volume than the treated tumors (*P <* 0.0047) Fig. 4e1-2. We also checked for the presence of carboplatin in the implanted films Fig. 4f and in the checked zones; with the help of electron microscopy and EDX, two zones were identified: zone 1 with few attached cells and zone 2 with many adherent cells. The presence of platinum was only in zone 1 Fig. 4g corresponding to the region of fewer attached cells, whiles no platinum was detected in zone 2 Fig. 4h corresponding to many attached cells.

#### Loss of cellular adherence and morphology

**Fig. 5:**
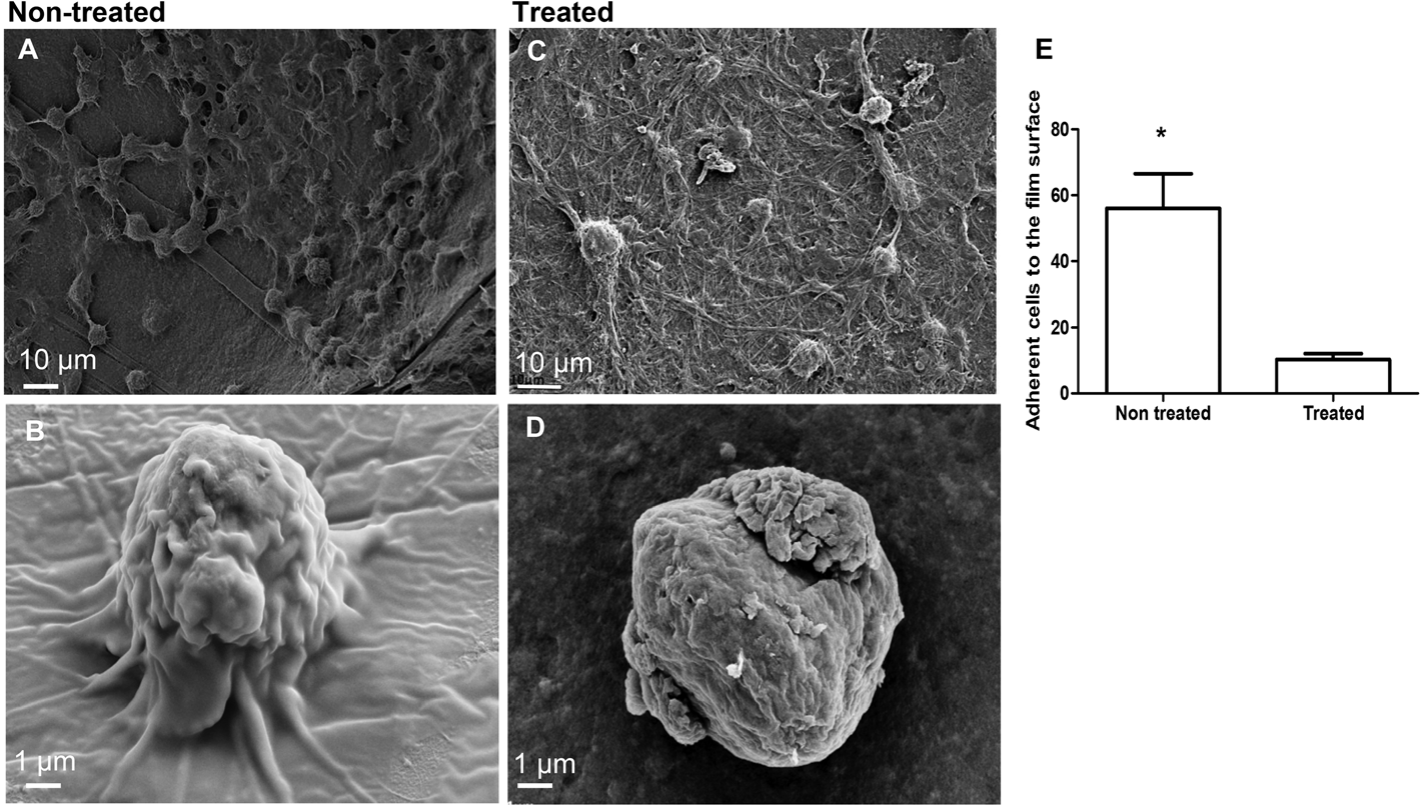
In vivo cell viability in the implanted films. **a**. Cells adhered well when the film was not treated. **b**. Higher magnification shows cell-adhering filopods with normal shapes. **c**. Few cells or cell residues are shown from the treated film. **d**. Higher magnification shows morphological changes and the loss of cell-adhering filopods. **e**. Histogram from three readings shows the difference in cell adherence rate in the control group compared with the nontreated group (*P* < 0.0131)

The implanted films of the control and treated samples were checked for cellular density and attachment to the film surface in the contact zone by SEM. Fig. 5a and b show cells with normal morphology and very good density in the attachment to the film. The presence of carboplatin led the cells to lose their filopods and exhibit an encapsulated stressed form Fig. 5c and d. Three regions were checked in both film types, and then the average cell number was calculated. Fig. 5e presents significantly fewer (five times) cells when the tumor was implanted with carboplatin-loaded film (*P <* 0.0131).

#### Histological study of implanted tumors

##### Allograft studies

**Fig. 6:**
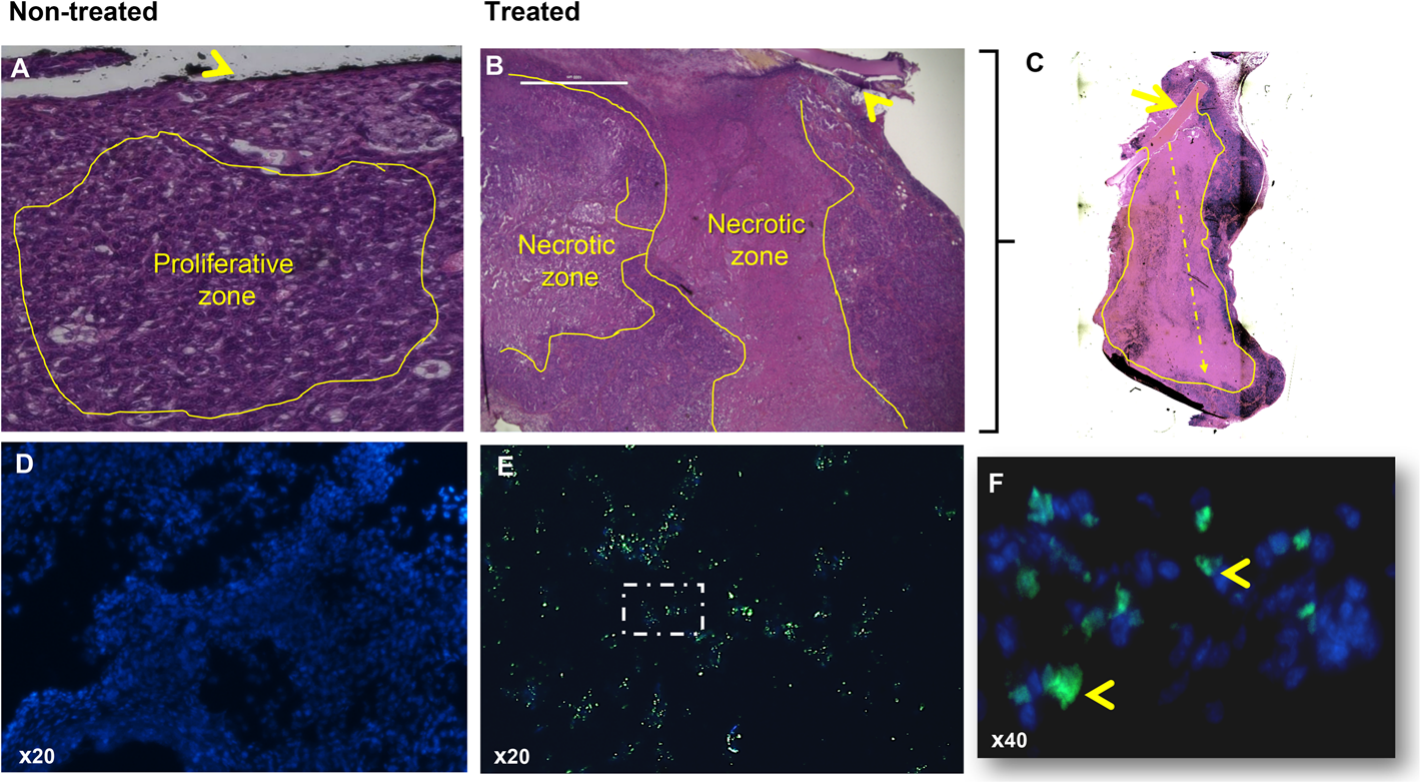
Apoptotic cell detection. Hematoxylin & eosin (H&E) coloration showing the proliferation zones (yellow drawing) in **a**. control and **b**. treated, necrotic zones detected where the film was in contact with the tumor. The black line in A and the yellow arrows represent the position of the implanted film **c**. Implanted tumor after 8 days of implantation. Pointed arrow represents the drug diffusion direction, yellow drawing represents the necrotic zone and yellow arrow indicates to the implanted film. Immunofluorescence check for the necrotic zone: **d**- control, there was no detected necrosis; **e**- Apoptotic parts were detected in the contact zone with the film; and **f**- Apoptotic zone at higher magnification. Blue represents nucleus, green represents apoptotic cells

Hematoxylin eosin coloration was performed to check the status of the CT26 tumors after implantation. Fig. 6a shows the control sample with active cancer cells, while necrotic zones were only noticed when the tumor was implanted with carboplatin-loaded film Fig. 6b. In Fig. 6a and b, the black line and yellow arrow over the control and treated samples indicate the position of the film during the implantation period. Fig. 6c shows the direction of the drug upon release from the film. To confirm the presence of the necrotic zone in the treated samples, both control and treated samples were tested via the apoptotic cell detection TUNEL method, where DNA fragments should be colored green. Fig. 6d shows the control sample without necrotic zones, and only the nuclei (blue) are colored. This is contrary to the treated samples in Fig. 6e and f, where dead cells are noted in green to represent DNA fragmentation. The measured necrotic zones were 4000 ± 169 μm.

##### Xenograft studies

**Fig. 7:**
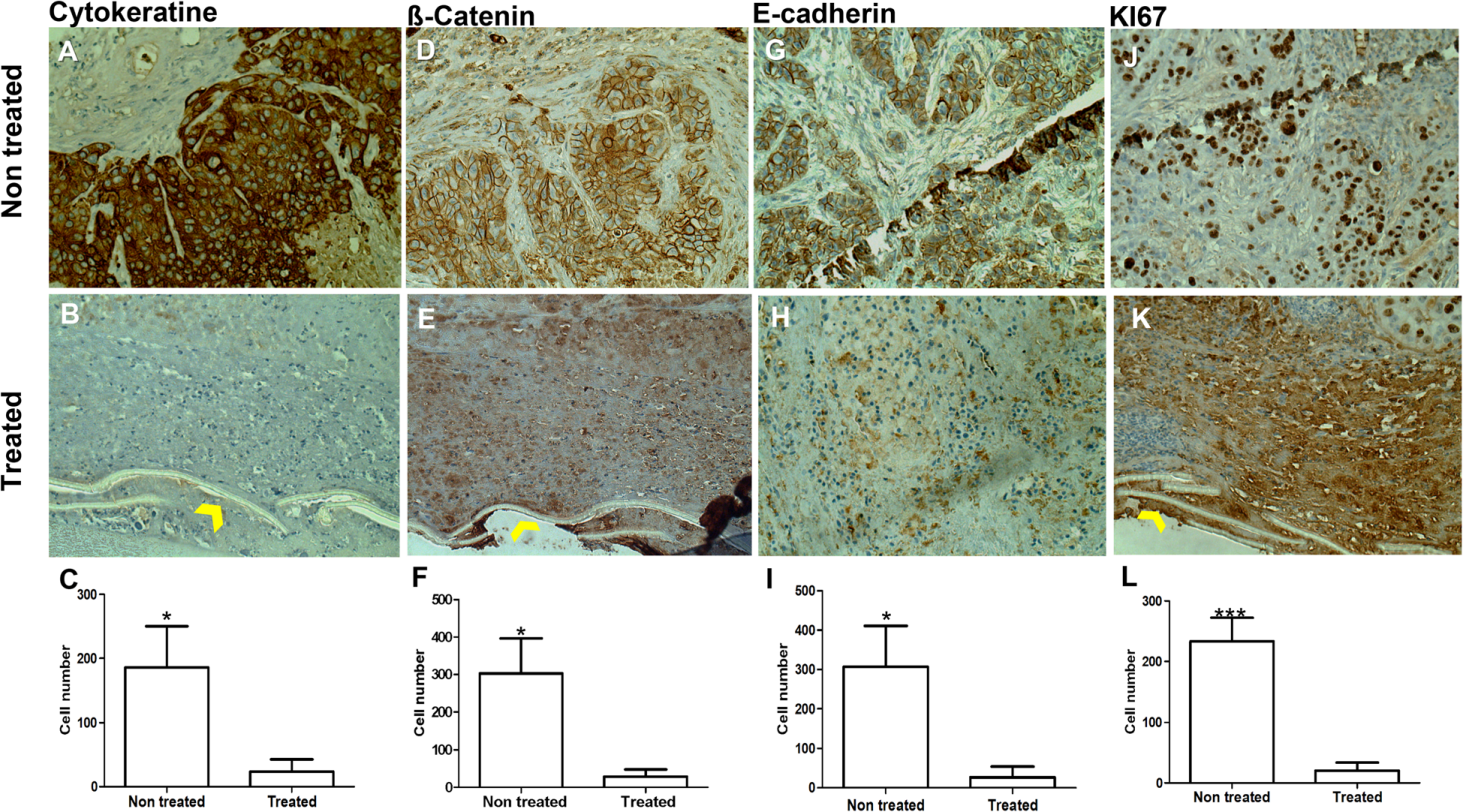
Morphological cell changes. Immunohistochemistry of the treated and control samples where **a**, **d**, **g** and **j** represent the tumor tissue implanted with the film without drug and are considered as the control. **b**, **e**, **h** and **k** represent tumor tissues with drug-implanted film. Yellow arrows in **b**, **e** and **k** treated group represent the degraded nanofilms **c**, **f**, **i** and **l** Histogram of five readings comparing the nontreated (NT) and treated zones (T). **a**, **b**, and **c**. Tissues treated with cytokeratin antibodies; **d**, **e** and **f**. Tissues treated with beta catenin antibodies; **g**, **h** and **i**. Tissues treated with E-cadherin antibodies; and **j**, **k** and **l**. Tissues treated with Ki67 antibodies

In a parallel experiment, OVCAR-3 cells (a human ovarian cancer cell line) were injected subcutaneously into nude mice. The implantation of nanofilms with or without carboplatin was achieved; 10 days later, the samples were embedded in paraffin. The slides produced (four microns) were treated with Ki67, E–cadherin, beta catenin and cytokeratin antibodies to study the proliferation and cellular adhesion of the cancer cells under both conditions (control and treatment). Fig. 7a, d, g, and j present the control samples, and Fig. 7b, e, h and k present the samples implanted with the films loaded with carboplatin. The cells lost their sharpness and attachment to each other, and little or no mitosis was detected in the zones affected by carboplatin. The yellow arrows in the Figures B, E and K represents the degraded nanofilms. Schematic graph of cancer cell – drug interaction, drug diffusion and film degradation are provided Fig. S3. A statistical study was performed for each antibody, including both the control and treated samples, via the *Mann*–*Whitney* test as shown in the histograms Fig. 7c, f, i and l for cytokeratin (*P <* 0.0212), beta catenin (*P <* 0.0294), E-Cadherin (*P* < 0.0265) and Ki67 (*P* < 0.0112). These antibodies show that cancer cell mitosis and activity were three times greater in the control samples than in the samples treated with carboplatin-loaded film.

## Discussion

In this study, we described the in vitro and in vivo local drug release efficacy produced via low-pressure plasma polymerized PCL-co-PEG polymers with encapsulated carboplatin loaded onto a collagen substrate. Our applied method of implanting the drug-loaded film into the tumor nodule based on nano biodegradable films showed a promising capacity to reduce the tumor size, killing cancer cells with no secondary effects registered in our BALB/c and nude mouse models. The films produced had a concentration of 300 μg of carboplatin encapsulated within a thickness of 200 to 600 nm on a 1 cm^2^ surface. The deposited membranes showed structural stability with or without the deposited drug; however, the in vitro incubation with culture medium showed some cracks on the film’s surface due to the swelling of the biodegradable collagen film during incubation and absorption of the culture medium Fig. S4.

The production of the tumor in the inguinal lymph node or subcutaneous administration helped us to technically fix the film and ensure that the contact zone of the deposited drug with the tumor remained the same.

We were able to identify the deposited drug via scanning electron microscopy (SEM), and no changes were noticed in the deposited zone position or the nature of the drug. Cancer cells were easily identified with their filopods adhering well to the copolymer. The measurement of the necrotic zone was in consideration with the implantation period of the films (10 days maximum). The length of treatment was chosen according to the ethical limitation of tumor growth in the mice and the release of carboplatin through the culture medium. A different protocol could be envisaged depending on the cancer cell line, the tumor model and the size at film implantation time.

Cancer surgery is based on simple rules, including obtaining free margin clearance in the case of tumor resection. However, tumors could be impossible to cure with free margins because they may touch major vessels or be an unresectable structure. Local recurrence risk after surgical resection is related to the presence or absence of margin involvement where a positive margin on a critical structure is a common situation, mainly for retroperitoneal sarcoma [26, 27]. Local treatment after surgery can be modeled using multilayered nanofilms to increase the rate of tumor control. Currently, no simple to use drug delivery system exists except after brain tumor resection with a specific sponge placed in the surgical zone [28, 29]. Taken together, in all these situations, the surgeon mainly identifies the place that is at risk of having a positive margin. In some cases, a metallic clip could be placed to identify the positive margin and then offered to a postoperative radiotherapy procedure. Here, we postulated that if the surgeon had a solution to deliver local chemotherapy of other drugs, it could be very effective. To be relevant, the device has to be i) well tolerated, including no adhesion to the small bowel or other fragile structures; ii) able to dissolve following drug release; and iii) able to deliver drugs completely to offer a unique treatment directed to a specific case (personalized medicine). Through this technology, drugs with different properties, including antibodies or nanocapsules, can be compiled with high concentrations according to the treatment protocol.

## Conclusion

Using radio frequency plasma technology, biodegradable multi-nanolayers with different thicknesses of the anticancer drugs deposited between the layers were designed, produced and used locally for cancer treatment in a mouse model. This combination can open another avenue for personalized medicine. Drugs with different properties, including antibodies or nanocapsules, can be compiled with high concentrations according to the treatment protocol.

## Supplementary information

**Additional file 1: Figure S1.** Schematic diagram of nanofilm generation using low-pressure inductively coupled plasma. A. Low-pressure inductively coupled plasma (ICP) reactor. B. Diagram of the copolymerization of PCL-PEG layers from monomers via radio frequency plasma.

**Additional file 2: Figure S2.** Position of the inguinal lymph node in mice model. White pointed rectangle and yellow arrow indicate the inguinal lymph node position on the abdominal wall of the mouse.

**Additional file 3: Figure S3.** Schematic diagram of fabricated nanolayers film composition and the mechanism of drug- cancer cell interaction. Schema represents the fabricated multi nanolayers composition and the mechanism of drug – cancer cell interaction through the implantation period and the degradation of the biodegradable film.

**Additional file 4: Figure S4.** Morphological changes to the film surface after in vitro incubation. A. Film without drug before incubation with culture medium. B. Film with drug before incubation with culture medium. C. After 1 day of incubation. D. After 7 days of incubation. E. Treated film after implantation and F. nontreated film after implantation; in both cases, there were no cracks on the surface but rather folds on the surface.

## Data Availability

The datasets used and/or analysed during the current study are available from the corresponding author.

## Abbreviations

ICP: Inductively Coupled Plasma
ICP-MC: Inductively Coupled Plasma Mass Spectrometry
PCL-PEG: Poly-ε-Caprolactone-Polyethylene Glycol
RF: Radio Frequency
SEM: Scanning Electronic Microscopy
OVCAR-3: Ovarian Cancer Cell Line
CT-26: Colon Cancer Cell Line
PBS: Phosphate Buffered Saline
RPMI: Roswell Park Memorial Institute
DMEM: Dulbecco’s Modified Eagle Medium
EDX: Energy-Dispersive X-ray spectroscopy
DAPI: 4′,6-Diamidino-2-Phenylindole
NT: Non-Treated
T: Treated
