# Supplementary figures and images for: Multi-nanolayer drug delivery using radiofrequency plasma technology
Source: BMC Cancer. 2020 Jun 17;20:565. doi: 10.1186/s12885-020-06989-w (PMC7302375; doi:10.1186/s12885-020-06989-w)

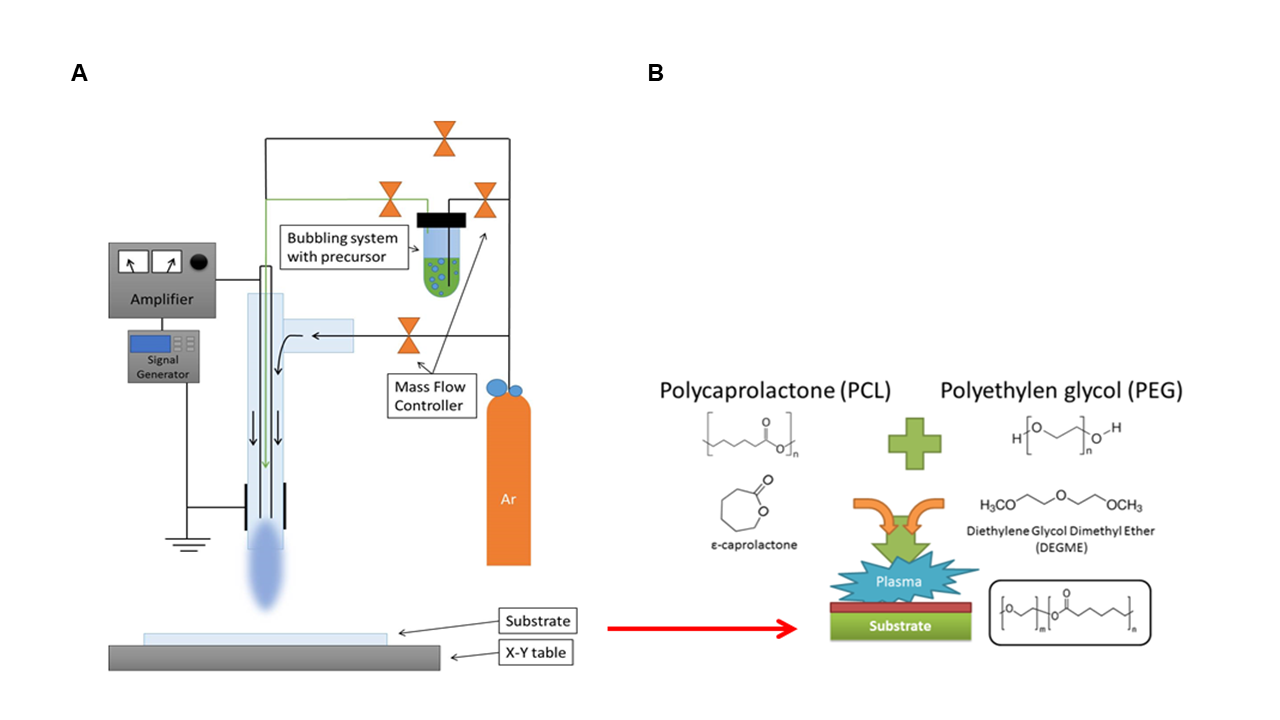

Supplement: Supplementary file 1 — Additional file 1: Figure S1. Schematic diagram of nanofilm generation using low-pressure inductively coupled plasma. A. Low-pressure inductively coupled plasma (ICP) reactor. B. Diagram of the copolymerization of PCL-PEG layers from monomers via radio frequency plasma. [file 12885_2020_6989_MOESM1_ESM.tif]

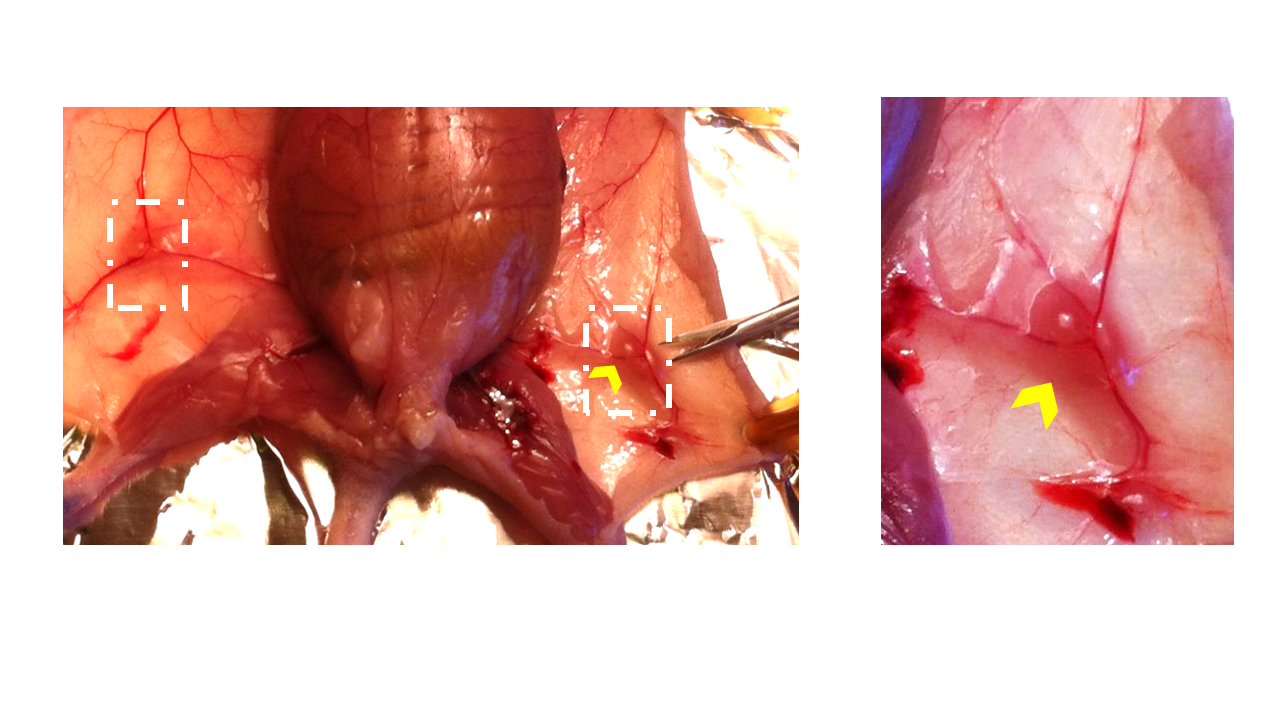

Supplement: Supplementary file 2 — Additional file 2: Figure S2. Position of the inguinal lymph node in mice model. White pointed rectangle and yellow arrow indicate the inguinal lymph node position on the abdominal wall of the mouse. [file 12885_2020_6989_MOESM2_ESM.tif]

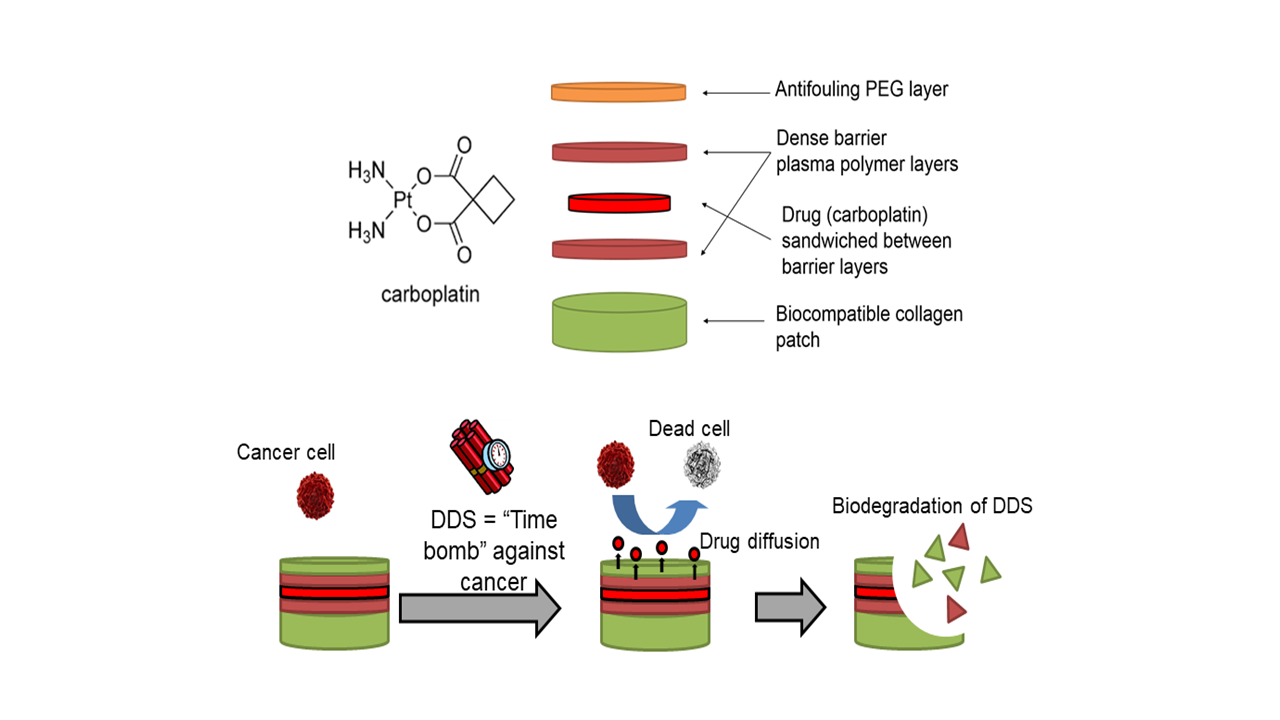

Supplement: Supplementary file 3 — Additional file 3: Figure S3. Schematic diagram of fabricated nanolayers film composition and the mechanism of drug- cancer cell interaction. Schema represents the fabricated multi nanolayers composition and the mechanism of drug – cancer cell interaction through the implantation period and the degradation of the biodegradable film. [file 12885_2020_6989_MOESM3_ESM.tif]

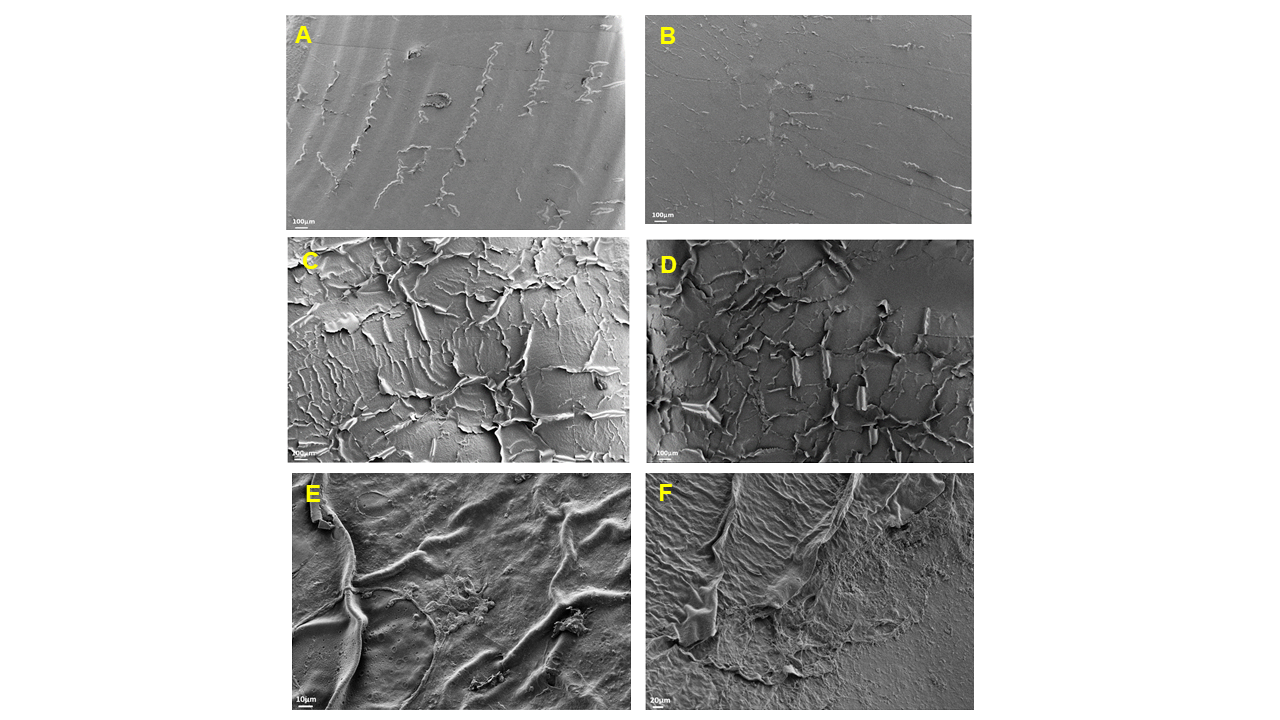

Supplement: Supplementary file 4 — Additional file 4: Figure S4. Morphological changes to the film surface after in vitro incubation. A. Film without drug before incubation with culture medium. B. Film with drug before incubation with culture medium. C. After 1 day of incubation. D. After 7 days of incubation. E. Treated film after implantation and F. nontreated film after implantation; in both cases, there were no cracks on the surface but rather folds on the surface. [file 12885_2020_6989_MOESM4_ESM.tif]
